# Report of the Pennsylvania Hospital for the Insane for the Year 1851

**Published:** 1852-04

**Authors:** 


Art. VI.—Report of the Pennsylvania Hospital for the Insane for the year 1851. By Thomas S. Kirkbride, M. D., Physician to the institution. Philadelphia: 1852.

This is an interesting report on the condition of one of the best managed institutions in our country—the eleventh by Dr. Kirkbride.

At the date of the last Report, there were 213 patients in the Institution; since which 204 have been admitted, and 201 have been discharged or died, leaving 216 under care at the close of the year.

The total number of patients in the Hospital during the year was 417. The highest number at any time was 243, the lowest was 206, and the average number under treatment during the whole period was 223.

For several months during the past year, the whole house was inconveniently crowded, but the general good health which then prevailed enabled us to receive all the cases that were brought to the Hospital, although much difficulty was often experienced in accommodating them. As a large proportion of the people of Pennsylvania have always been accustomed to look to the Pennsylvania Hospital for relief for their friends when afflicted with insanity, it was felt to be a duty, as far as possible, to receive all whose condition rendered their removal from home important, or who were likely to suffer from the want of prompt treatment. The prospect of the State provision for the insane being soon available, was also another reason for our being willing, for a short period, to be somewhat incommoded by too large a number of patients, and we now have reason to believe that our present apartments will hereafter prove ample to accommodate all applicants for admission.

Of the patients discharged during the year 1851, were

| | |
|---|---|
| Cured | 107 |
| Much improved | 13 |
| Improved | 32 |
| Stationary | 23 |
| Died | 26 |
| Total | 201 |

Of the patients discharged "cured," fifty-two were residents of the Hospital not exceeding three months; twenty-six between three and six months; twenty-three between six months and one year; and six for more than one year.

Of those discharged "much improved," three were under treatment less than three months; five between three and and six months; two between six months and one year; and three for more than one year.

Of the "improved," nine were under care less than three months; five between three and six months; eleven between six months and one year; and eight for more than one year.

Of those discharged and reported "stationary," six were under care less than three months; four between three and six months; four between six months and one year; and nine for a longer period than one year.

Sixteen males and ten females have died during the year. Of these deaths, eight resulted from acute mania; one from acute dementia; five were cases of organic disease (softening) of the brain, of which the mental affection was only a symptom; one was from epilepsy; two from pulmonary consumption; three from dysentery; one from chronic ulceration of the throat; one from the exhaustion produced by a long-continued refusal of food; one from suicide; one from cancer; and two from old age.

Of these deaths, eight occurred within less than a fortnight after their admission; while two were of patients who had been thirty-six years residents of the Hospital, and one had been more than twenty-five years.

Several of the cases of acute mania were undoubtedly injured by the journey to the Hospital during the existence of the acute symptoms, which often cannot, without difficulty, be distinguished from those of inflammation of the brain. While this doubt exists, the patient had better be retained at home, and the probabilities of ultimate recovery are not lessened by such a course.

Of the patients who died, ten were admitted for mania; six for melancholia; and ten for dementia.

Among the numerous resources of the Hospital for affording to the inmates not only comfort, but healthful and rational amusement, we have only room to allude to the *museums* and *reading rooms*, of which Dr. Kirkbride gives the following description:—

On Christmas day, 1848, this Hospital was presented with a handsome building, to be used as a Museum and Reading-Room, for the patients of the Institution. This building is situated 186 feet west of the centre building, on the south line of the open space on that side, and was erected, furnished, and supplied with a collection of books and curiosities without cost to the Institution, from the contributions of its officers and patients, and their friends, and of others who felt an interest in promoting the comfort of its inmates.

This Reading-Room has been in daily use since that time; occupied in the morning by the ladies, and in the afternoon by gentlemen; and has afforded great satisfaction and enjoyment to a large number of patients.

In my Annual Report to your Board, last year, I stated that the only drawback in the arrangement was the fact that the patients of each sex had the use of the building but the half of each day; so that often, when desiring to use it, the prescribed hours prevented. It was also suggested that the erection of a similar building in a corresponding point of the open space, exclusively for the gentlemen, so as to give up the one already in use entirely to the ladies, would be an admirable object for the active benevolence of some philanthropic individuals, anxious to confer an especial favor upon the insane of our community. It gives me great pleasure to be able to report, that this work has already been accomplished, and that our patients are now enjoying this additional means of occupation and amusement so promptly offered to them.

The suggestion above referred to had no sooner met the eye of one of our liberal and enlightened citizens than, appreciating its importance, he promptly tendered $100 in aid of the work, whenever it should be commenced. This liberal offer, and a letter from a number of the patients, induced your Board to authorize the writer to proceed with the erection of the building, provided none of the funds of the corporation were applied thereto. Soon after this, application for aid to the undertaking was made to a few of our benevolent citizens, who responded to the call in so liberal a manner, that the work was commenced early in the summer, and was finished and occupied before the close of the year.

This new structure, in size and external appearance, corresponds almost entirely with the Museum and Reading-Room previously erected, and the two now form a symmetrical feature in the arrangement of our buildings. It is one story high, 46 by 24 feet, built of stone, rough-cast externally, and is covered with slate. It is in one room, lighted by windows on the western side, and by two skylights in the roof, each of which are four feet square. The ceiling is 13 feet high to the square, and is groined to each skylight, so as to be 20 feet high at these points. A piazza, 8 feet wide, extends along the entire western front, beyond which is a private yard, intended to be handsomely improved. The building is warmed by a hot-water apparatus, and ample provision has been made for a forced ventilation. Cases have been placed on the eastern side of the room; a handsome specimen of stained glass is over each door; and a fine marble tablet in the western wall, gives the origin of the structure, which may be regarded as a memorial, from this Institution, of the First Centennial Anniversary of the Founding of the Pennsylvania Hospital.

The whole arrangement of the Reading-Room is such as to make it an attractive place of resort, especially to the convalescent, and the cultivated, studious patient; to all, indeed, who desire a cheerful and comfortable apartment, where they can quietly read and study, or amuse themselves by inspecting the various objects of interest, spread out before them.

The funds which have been contributed have not only enabled us to put up and furnish the building, but also to procure a very fair beginning for a library, and a foundation for a collection of specimens of natural history, birds, minerals, shells, &c., and some pictures and busts; to all of which classes of objects contributions are respectfully solicited, from those who are disposed to add to the means of rational amusement possessed by the patients of this Institution.

A fine Dioptric prismatic lantern and a microscope have also been procured from the same source.

The provision of these Reading-Rooms, in connection with collections of objects of interest, always kept ready for use, and entirely disconnected with the wards, although strictly private, cannot but be regarded as a progressive step in the treatment of the insane. They supply a want that has everywhere been felt by individuals of refined feelings and cultivated minds, especially when so far restored, as, although not ready to leave a hospital, are still quite well enough to be much annoyed by many incidents that are liable to occur in full wards. A multiplication of such and kindred means can scarcely fail to render a prolonged residence in an institution less irksome than it would otherwise be. They must be regarded as belonging to that class of remedies which we are only beginning to supply, but which, with the advance of knowledge and practical Christianity, will yet go far, it is to be hoped, to cause our hospitals for the insane to be considered, as well by patients as their friends, and the whole community, as simply places of resort, which all experience has shown to be necessary, in a majority of cases, for the relief of a distressing malady. Although it must be expected that a residence in them is to be coupled with some unavoidable restraint, attended with some privations, and not furnished with all the comforts and luxuries of every home, still it may be surrounded with so much that is cheering and attractive, so many, and such varied means of amusement and occupation, that what is unpleasant may well be forgotten, in the recollection and enjoyment of a restoration to health, to society, and to happiness, which have resulted from their use.

